# Structural and Oxidative Changes in the Kidney of Crucian Carp Induced by Silicon-Based Quantum Dots

**DOI:** 10.3390/ijms130810193

**Published:** 2012-08-16

**Authors:** Sorina Nicoleta Petrache, Loredana Stanca, Andreea Iren Serban, Cornelia Sima, Andreia Cristina Staicu, Maria Cristina Munteanu, Marieta Costache, Radu Burlacu, Otilia Zarnescu, Anca Dinischiotu

**Affiliations:** 1 Department of Biochemistry and Molecular Biology, University of Bucharest, 91-95 Splaiul Independentei, Bucharest 050095, Romania; E-Mails: sori.petrache@yahoo.com (S.N.P.); lory_stanca@yahoo.com (L.S.); cristinastaicu_bio@yahoo.com (A.C.S.); cristina_munteanu06@yahoo.ca (M.C.M.); marietacostache@yahoo.com (M.C.); otilia_zarnescu@yahoo.com (O.Z.); 2 Department of Preclinical Sciences, University of Agricultural Sciences and Veterinary Medicine, 105 Splaiul Independentei, Bucharest 050097, Romania; E-Mail: irensro@yahoo.com; 3 Laser Department, National Institute of Laser, Plasma and Radiation Physics, 409 Atomistilor, Bucharest-Magurele 077125, Romania; E-Mail: simac@ifin.nipne.ro; 4 Department of Mathematics, University of Agricultural Sciences and Veterinary Medicine, 59 Marasti Bd., Bucharest 011464, Romania; E-Mail: r.burlacu@usamv.ro

**Keywords:** quantum dots, fish kidney, oxidative stress

## Abstract

Silicon-based quantum dots were intraperitoneally injected in *Carassius auratus gibelio* specimens and, over one week, the effects on renal tissue were investigated by following their distribution and histological effects, as well as antioxidative system modifications. After three and seven days, detached epithelial cells from the basal lamina, dilated tubules and debris in the lumen of tubules were observed. At day 7, nephrogenesis was noticed. The reduced glutathione (GSH) concentration decreased in the first three days and started to rise later on. The superoxide dismutase (SOD) activity increased only after one week, whereas catalase (CAT) was up-regulated in a time-dependent manner. The activities of glutathione reductase (GR) and glutathione peroxidise (GPX) decreased dramatically by approximately 50% compared to control, whereas the glutathione-S-transferase (GST) and glucose-6-phosphate dehydrogenase (G6PDH) increased significantly after 3 and 7 days of treatment. Oxidative modifications of proteins and the time-dependent increase of Hsp70 expression were also registered. Our data suggest that silicon-based quantum dots induced oxidative stress followed by structural damages. However, renal tissue is capable of restoring its integrity by nephron development.

## 1. Introduction

Quantum dot (QD) semiconductors are special nanoparticles possessing optical properties that have elicited the interest of scientists in imaging studies in biology and medicine [1], as well as the development of vectors for drug delivery [2]. These nanoparticles have fluorescent properties with broadband excitation, narrow bandwidth emission, high intensity of emitted light and good photostability [3]. Such characteristics are the reason for their use as fluorescent probes for detecting specific biomarkers *in vitro*, at the cellular level, and *in vivo*, at the tissue and whole-organism level. For these applications, QDs have to possess a degree of biocompatibility. In this context, a major downfall of QDs is that they are usually composed of toxic elements such as Te, Se and Cd. In order to protect the biological microenvironment, it is possible to mask the cytotoxic core and/or shell of QDs by encapsulation in amphiphilic polymers [4] or in liposomes [5], coordinated with specific ligands; this can be used for focused targeting of a cell-type of interest, thus allowing for either localized administration of a therapeutic drug or imagistic techniques [1].

Because QDs are ideally used for imaging live cells and organisms over long periods of time, they have to present low toxicity. The cytotoxicity of QDs is dependent on a large number of factors including their size, the core and active surface composition, the type of encapsulation and processing parameters of nanoparticle synthesis, as well as the concentration of QDs being used [6]. Several QD toxicity studies have been done by *in vitro* and *in vivo* studies. Although recent studies have mainly employed the mouse and rat animal models, freshwater and marine fish have become popular models for toxicological research for several diseases, such as cancer, diabetes, muscular dystrophy, *etc*. [7], as well as for imaging studies [8].

Bony fish kidney is one of the first organs damaged by water pollutants [9,10]. Tubule degeneration, dilatation of glomerulus capillaries and changes of Bowman’s space are the most common structural changes [9], but they are not specific to the type of the xenobiotic. Unlike mammals, fish kidney is designed for regeneration and *de novo* nephron development [10,11]. Injured cells of the urinary tubules are replaced by new epithelial cells in an effort to restore structural integrity.

Once within the cells, QDs can release heavy metal ions, generate reactive free radicals [12] and interact with cellular components [13].

The problem of QD toxicity is controversial and might be overcome by using silicon-based QDs, which seem to have reduced toxicity [14,15].

The aim of this study is to investigate the *in vivo* renal effects induced after the intraperitoneal (IP) injection of a silicon-based fluorescent nanoparticle suspension in *Carassius auratus gibelio*.

The bio-distribution of Si QDs after IP injection was visualized by fluorescence imaging. The effects of oxidative stress were evaluated by analysis of malondialdehyde (MDA), carbonyl derivatives, thiol proteins and advanced oxidation protein products and reduced glutathione (GSH), as well as Hsp 70 expression. In addition, a set of characteristic parameters was determined, such as the activities of the antioxidant enzymes: superoxide dismutase (SOD), catalase (CAT), glutathione peroxidase (GPX), glutathione reductase (GR), glutathione-*S*-transferase (GST), glucose 6-phosphate dehidrogenase (G-6-PDH) after one, three, and seven days of treatment.

## 2. Results and Discussion

### 2.1. Microscopy Studies

Most of the work on tissue distribution of nanoparticles has been restricted to rodents [16–19]. To date, the only studies about the toxicity of silver [20] and titanium dioxide nanoparticles have been performed on fish [21]. The present study, as far as we know, is the first report describing silicon-based QD distributions in the fish kidney. Previous studies on mice have indicated that silica nanoparticles mostly accumulate in the organs of the reticuloendothelial system, such as the liver and spleen, and also in the kidney [17–19,22].

The IP injected QDs were taken up by blood circulation of *C. auratus gibelio* and spread in tissues such as the white muscle [14] and kidney.

**Figure 1:**
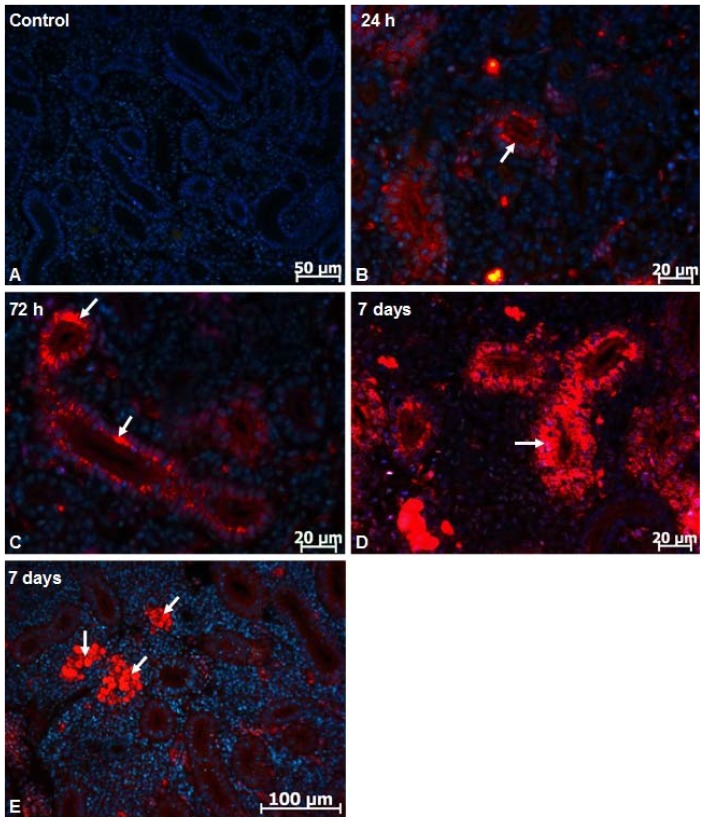
Silicon-based QD localization in the kidney of *Carassius auratus gibelio*. (**A**) Control (non-injected) animals. Visualization of silicon-based QDs in the different nephron segments at 24 h (**B**); 72 h (**C**) and 7 days (**D**,**E**) after IP injection. Note the progressive loading of renal tubular epithelial cell with silicon-based QDs (arrows). Arrows in figure E indicate macrophage clusters.

Due to intrinsic photoluminescence under ultraviolet excitation, silicon-based QDs have been detected in tissue sections by the emission of red light (Figure 1A–D). The fluorescence was not detectable for the control (non-injected and injected with NaCl 7‰) animals (Figure 1A). At 24 h after IP injection, silicon-based QDs were detected mainly in the epithelial cells cytoplasm of the proximal tubule (Figure 1B). At 3 (Figure 1C) and 7 days (Figure 1D) after IP injection cells from proximal tubule, distal tubule and collecting tubule were loaded with silicon-based QDs. The density of silicon-based QDs in the cytoplasm of cells from different nephron segments was higher at 3 and 7 days after IP injection. In the interstitial tissue, fluorescent silicon-based QDs were detected in the macrophages (Figure 1E).

**Figure 2:**
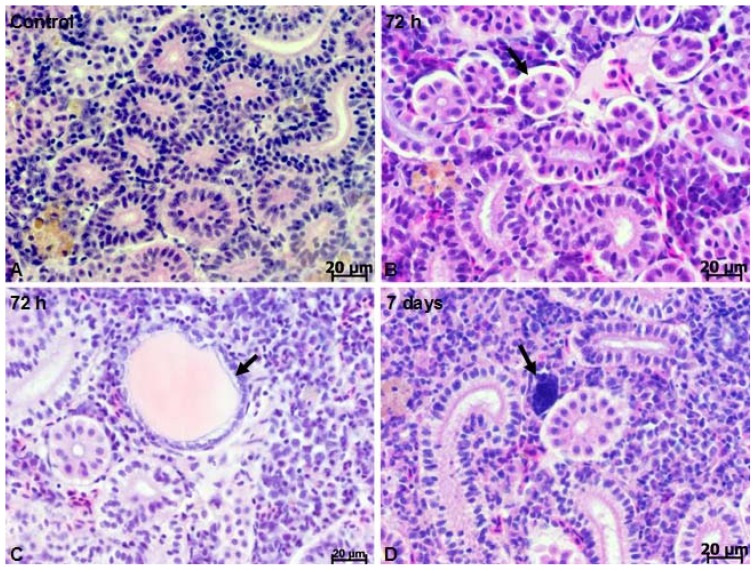
Kidney histology of *Carassius auratus gibelio*. (**A**) Control (non-injected) animals; (**B**) histopathology of crucian carp kidney at 72 h after IP injection indicates detached epithelial cells from basal lamina (arrow) and dilated tubules; (**C**) dilated tubule (arrow) in the crucian carp kidney at 72 h after IP injection; (**D**) basophilic cluster (arrow) adjacent to damaged renal tubules. H&E staining.

To investigate the toxicity, histological assessment was performed to determine whether silicon-based quantum dots cause kidney damage. The crucian carp trunk kidney is composed of nephrons and collecting ducts surrounded by interstitial tissue (Figure 2A). In the kidney of fish injected with silicon-based QDs alterations were observed, including detached epithelial cells from basal lamina (Figure 2B), dilated tubules and debris in the lumen of tubules (Figure 2C). Besides degenerative processes, at 7 days after IP injection, kidney regeneration was also observed in *Carassius auratus gibelio*, indicating that silicon-based QDs do not impede the regeneration of tissue. Nephroneogenesis was characterized by the appearance of small, intensely basophilic clusters of cells, frequently near collecting ducts (Figure 2D).

By contrast to marine fish species, the glomerulus of freshwater fish is well vascularized and the glomerular filtration rate is high. Freshwater fish produce large amounts of dilute urine, maintaining the acid-base and electrolyte equilibria and regulating the blood pressure.

However, at the level of the renal corpuscle, in the absence of the glomerular basement membrane and podocytes, the sole dividing barrier between the mesangium and glomerulus is the fenestrated endothelium. Due to the high rate of glomerular filtration, nanoparticles smaller than 10 nm in diameter diffuse and accumulate in the mesangium after they depart from the glomerulus through these pores [23].

Histological examination revealed that the kidney of the crucian carp was able to regenerate after silicon-based QD injury. The formation of clusters of basophilic cells represents the first phase of *de novo* nephroneogenesis in fish kidney, followed by the development of a cavity which grows to form the renal vesicle and later on glomerulus and Bowman’s vesicle [24]. Previous studies have indicated that chemically-induced nephrotoxicosis in fish is followed by new nephron development, and tubular regeneration can be a good sign of adaptation and recovery [25]. This process was observed in the kidney of many fish species, including goldfish [26], rainbow trout [27], zebrafish [24], tilapia [11] and medaka [28].

The histological studies revealed that in addition to degenerative processes visible at 7 days after IP injection, kidney regeneration could also be observed in *Carassius auratus gibelio*, indicating that silicon-based QDs do not impede tissue regeneration, *i.e.*, nephroneogenesis.

The detection of QDs in the macrophages might explain the increased CAT and SOD activities after 3 and 7 day exposure; this could be explained by the fact that these cells, when activated, produce oxygen and nitrogen reactive species during phagocytosis [29]. In theory, the contact with silicon QDs could generate the ROS cascade which ensues, either due to the activity of the macrophage NADPH oxidase (NOX) complex, or due to the activation by mechanical stress of NADPH oxidase expressed by renal cells. Taking into account that the greatest quantity of QDs is present in the renal tubular epithelial cells it seems that these could contribute to a larger extent to superoxide anion generation. In the kidney, NOX1 and NOX4 are present [30], but only NOX4 is highly expressed [31].

### 2.2. Biochemical Studies

#### 2.2.1. Lipid Peroxidation

The MDA level increased by 97% after one day of exposure, reaching the maximum level of 288% higher than the control after the third day, as indicated in Table 1. The doubling of MDA concentration was registered as a consequence of the cascade mechanism of lipid peroxidation. Taking into account that GPX activity decreased after three and seven days, whereas GST activity significantly increased during the same period, it would seem that GST was involved in the decrease of MDA after 7 days following its conjugation with GSH.

The decrease of MDA concentration after 7 days could also be explained by the significant increase of SOD and CAT activities, which are involved in ROS elimination.

#### 2.2.2. GSH Level

GSH plays a central role in antioxidant defense and in the regulation of several signal transduction pathways and metabolic functions [32], and is considered the most important antioxidant *in vivo*. The GSH concentration (Table 1) decreased by 66%, 60% and 47% respectively after 1, 3 and 7 days of treatment, compared to controls. As these data indicated, the response of the kidney cells to QD exposure initially involved a decrease of the GSH level, as it was probably consumed by indirect chemical reactions, as well as GPX and GST catalyzed reactions that removed the deleterious compounds.

This tri-peptide is synthesized intracellularly from glutamate, cysteine and glycine in the proximal tubular cells and can remain in the cell or be exported in the extracellular space. In the cell, the most important part of GSH is present in the mitochondrial pool, the rest being located in the cytoplasm [33]. In our case, the GSH formation was not sustained by enzymatic reduction catalyzed by GR, but, possibly due to *de novo* synthesis, induced by lipid peroxidation products [34]. This could be the reason why the GSH concentration increased by 56% after the seventh day, compared to the first day of exposure. Our data indicate that in the first three days of exposure, the decrease of the GSH concentration is in accordance with the rise of MDA level; conversely, the decrease of MDA concentration after the seventh day can also be explained by the increase of the GSH concentration.

#### 2.2.3. Oxidative Protein Alterations

ROS that elude the antioxidant system attack the proteins. Sulfur and aromatic-containing amino acid residues, such as tyrosine, cysteine and methionine are particularly susceptible to oxidative transformation [35]. Advanced oxidation protein products (AOPP) are formed *in vivo* as a consequence of the exposure of proteins to hypochlorous acid generated by myeloperoxidase, by oxidation of tyrosyl and histidyl residues, as well as by the deamination of lysyl residues [36]. These oxidative markers correlate with the levels of dityrosine [37] and could reflect the QD’s exposed macrophage involvement in the oxidative stress in fish kidney. AOPP concentration (Table 2) increased significantly after 3 days compared to control, whereas after 1 and 7 days, a non-significant difference of approximately 10% was observed.

In the case of cysteine, oxidation leads to the formation of disulfide bonds, mixed disulfides (e.g., with glutathione) and thiyl radicals [38]. The protein thiol level (Table 2) decreased significantly by 28%, 33% and 43% respectively after 1, 3 and 7 days of treatment, compared to control.

Our results suggest that the dityrosyl cross-links appeared due to the ROS attack on proteins could be lowered by the intervention of protein cysteine residues.

Carbonyl derivatives of proteins are formed by ROS-mediated oxidation of the side chains of some amino acid residues (especially proline, arginine, lysine, threonine), as well as by a reaction with glucoxidation and lipid peroxidation products [39]. Reactive carbonyl compounds derived from lipids and carbohydrates react with proteins resulting in a “carbonyl stress” which generates kidney pathologies [40]. The protein reactive carbonyl group level (Table 2) was significantly up-regulated after 3 and 7 days of exposure by 122% and 115% respectively compared to control levels. In our case, the excess MDA could react mainly with amino groups of lysine residues, producing MDA-modified protein adducts.

Protein oxidation has been associated with renal damage. The radicals generated on tyrosine could migrate to the cysteine residues, forming a thiyl radical on a local cysteine residue, which can generate a very reactive sulfenic acid; the latter might be converted back to the reduced state or, alternatively, further oxidized, depending on the presence of additional redox active molecules [41]. The downregulation of protein thiols could be due to intra-protein or inter-protein disulfide formation that affects the protein tridimensional structure and function. But, in some cases, these alterations do not cause long time changes in protein function, because tioredoxin or glutaredoxin rapidly catalyze the reduction of disulfide bonds.

However, considering the mild histological modifications, we could conclude that silicon-based QDs induced the formation of oxidized proteins to a moderate degree, which could be degraded by the proteosomal system, as was proved by previous studies [42], avoiding the cross-linking aggregation and installment of possible pathological situations.

#### 2.2.4. The Antioxidant Scavenging Enzymes

**Figure 3:**
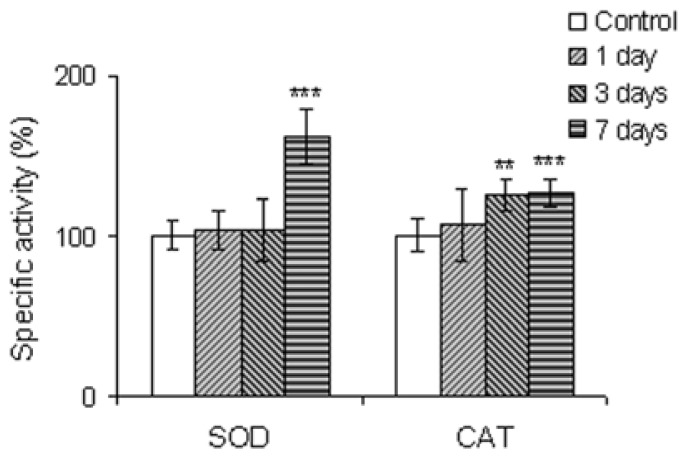
SOD and CAT relative specific values in kidney cells of *Carassius auratus gibelio* IP injected with silicon-based QDs up to seven days of exposure. Data are calculated as means ± SD (*n* = 6 in each group at each time point) and expressed as % from time point controls; * *p* ≤ 0.05, ** *p* ≤ 0.01, *** *p* ≤ 0.001.

The SOD activity was unchanged in the first three days of exposure, but in the seventh day, an increase of 56.5% was observed, whereas CAT activities increased in a time-dependent manner, by 25% and 27% after 3 and 7 days of exposure, respectively (Figure 3).

#### 2.2.5. Enzymes Involved in Glutathione Metabolism and in Generating Reducing Equivalents

After the first day of exposure, the increase of SOD and CAT activities suggests an insignificant increase of superoxide level and its downstream metabolite, hydrogen peroxide. When the concentration of hydrogen peroxide increased after the third and seventh days, catalase started to catalyze its degradation. After the seventh day, SOD activity increased significantly and the dismutation of superoxide occurred. Our results are in agreement with those of Oruc *et al.* (2004) who studied the SOD activity changes in kidneys of *Oreochromis niloticus* and *Cyprinus carpio* exposed to pesticides [43]. Nevertheless, in the presence of various doses of cadmium, a very toxic xenobiotic, this activity significantly decreased in the kidney of *Clarias gariepinus* [44]. Taking into account that catalase specific activity changed by a maximum of 27% after seven days of exposure, it seems that the generation of superoxide anions was at a moderate level.

**Figure 4:**
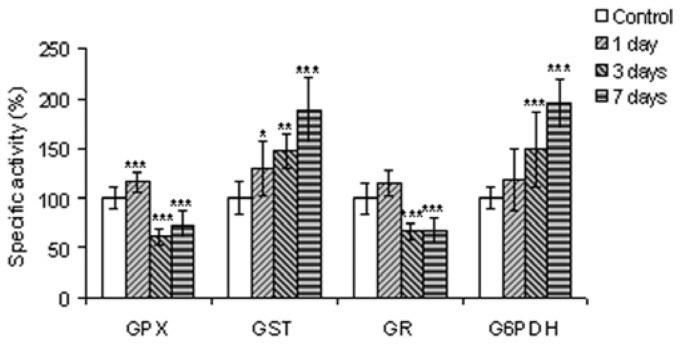
GPX, GST, GR and G6PDH relative specific values in kidney cells of *Carassius auratus gibelio* IP injected with silicon-based QDs up to seven days of exposure. Data are calculated as means ± SD (*n* = 6 in each group at each time point) and expressed as % from time point controls: * *p* ≤ 0.05, ** *p* ≤ 0.01, *** *p* ≤ 0.001.

The activities of GR and GPX increased after one day of exposure by 14% and 16% respectively, but decreased dramatically after 3 and 7 days by approximately 50% compared to control (Figure 4). The decrease of GR specific activity after the third day of exposure to QDs generated a decreased formation of reduced glutathione, and probably a lower level of protein glutathionylation. Additionally, the GST activity increased by 29.32%, 47.48% and 87.64% after one, 3 and 7 days of treatment, respectively (Figure 4).

The profile of GPX specific activity is inversely proportional with that of catalase. One possibility would be that, in the first day, the very low quantity of hydrogen peroxide was decomposed by GPX. At longer exposure times, this enzyme was not efficient in both hydrogen peroxide as well as lipid detoxification of peroxidation products. The variation of GST activity increased continuously, suggesting a significant role in the detoxification process in fish kidney, with some GST isoforms having a selenium independent GPx activity. This result is in agreement with previous findings [45].

The specific activity of G6PDH increased significantly by 49% and 94% after 3 and 7 days of treatment, respectively, compared to controls (Figure 4). This enzyme catalyzes the oxidative branch of the pentose phosphate pathway, generating NADPH, an electron donor in reductive biosynthesis, which is used for GSH regeneration [46]. According to our results, it appeared that NADPH was used for GSH regeneration at a reduced rate and only on the first day. It is also possible that this cofactor could interact directly with free radicals and have, after seven days, a pro-oxidant action being aimed at superoxide generation in the reactions catalyzed by NOXs.

### 2.3. Heat Shock Protein Hsp 70 Evaluation

**Figure 5:**
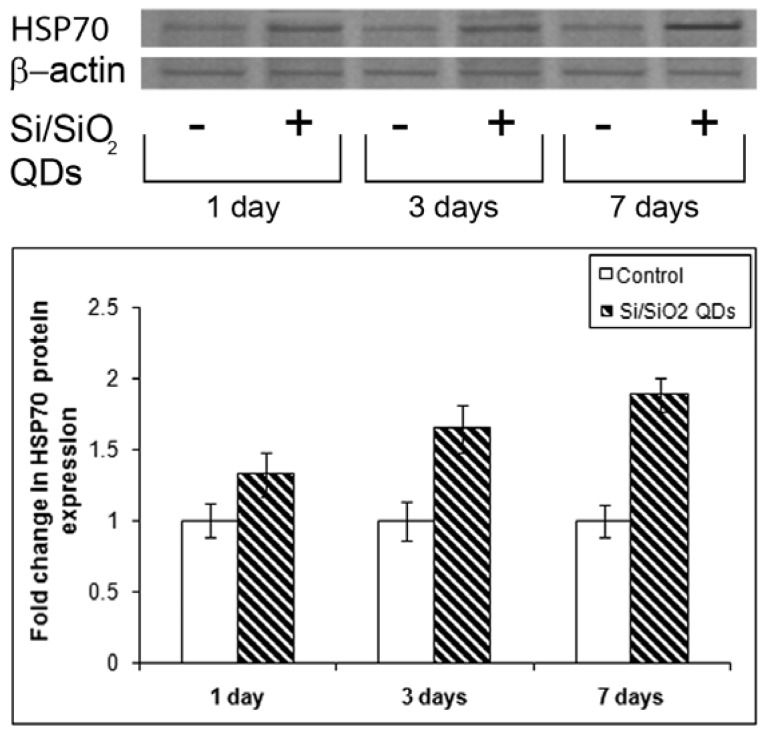
Western Blot analysis of heat shock protein Hsp 70 in the kidney after IP injection with silicon-based quantum dots (2 mg/kg body weight).

Fish kidney tissue as well as liver and gill are sensitive to Hsp response [47]. The Hsp 70 family is highly conserved and generally acts under stress conditions [48]. Our results have shown that Hsp 70 protein expression in the kidney of IP injected fish with Si/SiO_2_ QDs increased in a time-dependent manner: 1.33-, 1.65- and 1.88-fold, after 1, 3 and 7 days, respectively, compared to controls (Figure 5).

These proteins assist, in an ATP-dependent manner, the folding of nascent proteins, the refolding of damaged proteins and mediate the degradation of irreversibly denatured proteins [49]; in addition, they also protect cells against caspase dependent and independent apoptosis [50]. It was also suggested that Hsp 70 forms mixed disulfides with other cytoplasmic proteins, conferring in this way a protection to redox sensitive proteins from potentially irreversible disulfide bond formation [51].

It seems that following the perturbation of the intracellular environment due to the exposure of kidney to silicon-based QDs, the resulting biochemical effects were not completely scavenged by detoxification processes and modifications at protein levels occurred. Hsp70 probably targeted the denatured proteins for degradation, which ensured kidney resistance, as previously thought [52].

The increase in Hsp 70 expression in fish exposed to different types of xenobiotics was also shown in other studies [53,54]. Our results are in agreement with recent literature [55] which revealed that IP injection of titanium dioxide nanoparticles in mice was associated with very light lesions in the kidneys.

## 3. Experimental Section

### 3.1. Chemicals

Nicotinamide adenine dinucleotide phosphate disodium salt (NADP^+^), nicotinamide adenine dinucleotide phosphate reduced tetrasodium salt (NADPH) and malondialdehyde tetramethyl acetal were supplied by Merck (Darmstadt, Germany). The Detect X^®^ Glutathione Colorimetric Detection Kit was purchased from Arbor Assay (Michigan, USA), polyvinylidene fluoride (PVDF) membrane from Sigma-Aldrich (St. Louis, MO, USA). Anti Hsp 70 and anti β actin mouse monoclonal antibody (IgG1) and were obtained from Acris Antibodies GmbH. Other chemicals used were of analytical grade and were from Sigma (St. Louis, MO, USA).

### 3.2. Nanoparticles

The SiO_2_/Si nanoparticles used in this study were obtained through laser ablation by the Laser Department of the National Institute of Laser, Plasma and Radiation Physics, Bucharest-Măgurele, Romania [56]. The particles are spherical with a crystalline Si core covered with an amorphous SiO_2_ layer (1–1.5 nm thick). The size distribution estimated from transmission electron microscopy image statistics was a lognormal function, in the range 2 to 10 nm, with the arithmetic mean value of the diameter of about 5 nm. The photoluminescence emission [57] measured at room temperature reaches maximum intensity at ~690 nm (~1.8 eV). For the experiments we used a suspension of nanoparticles (2 mg/mL) prepared in 0.7% NaCl.

The SiO_2_/Si nanoparticles were produced by laser ablation method [58,59]. Briefly, SiO_2_/Si was synthesized in a stainless steel chamber initially evacuated to a base pressure of 4 × 10^−6^ mbar by a turbo-molecular pump. High purity (99.99%) helium flowed continuously, with a flow rate of 1L/minute, maintaining the pressure in the chamber at 550 mbar. The laser beam is focused onto a silicon target (energy density 8 J/cm^2^) placed inside of the chamber. Following laser-target interaction, the nanoparticles were ejected and driven by the flowing gas towards a Millipore filter (100 nm pores, placed at a distance of 5 cm from the target). Due to the Van der Waals and electrostatic forces, the nanoparticles deposit onto the filter surface without passage through the pores. After a reasonable quantity of nanopowder is collected, the filter is removed from the chamber. The silicon nanoparticles react with the oxygen from air and a silicon oxide layer is formed onto the nanoparticle surfaces, resulting in SiO_2_/Si. A Nd:YAG laser (355 nm wavelength, 5 ns pulse duration, 10 Hz repetition rate, with 60 mJ energy/pulse) was used. For investigation of SiO_2_/Si nanoparticles, a transmission electron microscope was used (model Philips, model CM 120, 200 kV).

The SiO_2_/Si particles are spherical with a crystalline Si core covered with an amorphous SiO_2_ layer (1–1.5 nm thick) [57]. The size distribution estimated from transmission electron microscopy image statistics was a lognormal function, in the range 2 to 10 nm, with the arithmetic mean value of the diameter of about 5 nm. The photoluminescence emission measured at room temperature reaches the maximum intensity at ~690 nm (~1.8 eV) [57]. For the experiments we used a suspension of nanoparticles (2 mg/mL) prepared in 0.7% NaCl.

### 3.3. Fish Maintenance and Treatments

Freshwater goldfish, *Carassius auratus gibelio,* with a weight of 90 ± 10 g and a length of 13 ± 2 cm, respectively, were acquired from The Nucet Fishery Research Station, Romania. The fish were acclimatized to laboratory conditions for three weeks prior to the experiment. Water quality characteristics were determined. The mean values for tested water qualities were: temperature 19 ± 2 °C, pH (7.4 ± 0.05), dissolved oxygen 6 ± 0.2 mg/L (constant aeration) and total hardness as CaCO_3_ 175 mg/L. The fish were maintained in a photoperiod with 12 h light/12 h dark. Feeding of pellet food at a rate of 1% of the body weight per day was stopped two days before initiation of the experiment, and no food was supplied to the fish during the experimental period.

Animal maintenance and experimental procedures were in accordance with the *Guide for The Use and Care of Laboratory Animals* (European Communities Council Directive 1986), and efforts were made to minimize animal suffering and reduce the number of specimens used. After the acclimatization period, the fish were randomly divided into three groups of ten individuals each, and placed in separate glass aquaria (250 L). The first group of fish was maintained in de-chlorinated tap water as a control. The second group was formed by fish intraperitoneally (IP) injected with NaCl 0.7% solution and third one was IP injected with 2 mg QDs/kg body weight. No mortality was noticed during the experiment. After one, three, and seven days from the injection of QDs, six fish from each group were cervical dislocated under light ether anesthesia and dissected to draw the renal tissue. Kidney samples were immediately frozen in liquid nitrogen and stored at −80 °C until analyses were performed.

### 3.4. Histology

Fragments of crucian carp trunk kidney were fixed in Bouin solution or 4% paraformaldehyde in PBS, dehydrated in ethanol, cleared in toluene and embedded in paraffin. 6 μm-thick sections were used for hematoxylin-eosin staining (H&E) and fluorescence microscopy.

### 3.5. Fluorescent Image Analysis of Nanoparticles Distribution

After deparafination and rehydration, slides were stained with 4,6-diamidino-2-phenylindole (DAPI) solution, mounted in PBS and analyzed by epi-fluorescence microscopy using a DAPI/FITC/Texas red triple band filter set (Zeiss). The photomicrographs were taken with a digital camera (AxioCam MRc 5, Carl Zeiss) driven by Axio-Vision 4.6 software (Carl Zeiss).

### 3.6. Preparation of Tissue Homogenates

Homogenates (prepared as 1 g of tissue per 10 volumes of buffer ) of fish kidney were prepared in ice-cold buffer (0.1 M TRIS-HCl, 5 mM EDTA buffer, pH 7.4) using a Mixer Mill MM 301 homogenizer. The resulting homogenate was centrifuged at 10,000 rpm for 30 min at 4 °C. The supernatant was aliquoted and stored at −80 °C and subsequently used for all types of analyses.

### 3.7. Biochemical Analysis

#### 3.7.1. Glutathione Assay

The total protein extract, deproteinated with 5% sulfosalicylic acid, was analyzed for total glutathione and oxidized glutathione (GSSG) using the Detect X^®^ Glutathione colorimetric detection kit and following manufacturer’s instructions. GSH concentration is obtained by subtracting the GSSG level from the total glutathione. The total and GSH levels were calculated as nmoles/mg protein.

#### 3.7.2. Malondialdehyde Assay

MDA, as an *in vitro* marker of lipid peroxidation, was assessed by a fluorimetric method [60]. To 200 μL of sample with a protein concentration of 4 mg/mL, 700 μL of 0.1 M HCl was added and the mixture was incubated for 20 min at room temperature. Then, 900 μL of 0.025 M thiobarbituric acid (TBA) was added and the mixture was incubated for 65 min at 37 °C. Finally, 400 μL of Tris-EDTA protein extraction buffer was added. The fluorescence of MDA was recorded using a Jasco FP750 spectrofluorometer with a 520/549 (excitation/emission) filter. A calibration curve with MDA in the range 0.05–5 μM was used to calculate the MDA concentration. The results were expressed as nmoles of MDA/mg protein.

#### 3.7.3. Protein Sulfhydryls Assay

The protein sulfhydryl concentration was calculated according to a method using 4,4′-dithiodipyridine (DTDP) [61]. A volume of 100 μL of total protein extract was mixed with 100 μL ice-cold 20% trichloroacetic acid (TCA) and vortexed. After 10 min on ice, the sample was centrifuged for 10 min at 10,000 rpm at room temperature. The supernatant was discarded and the pellet was rendered soluble in 20 μL of 1 M NaOH and mixed with 730 μL of 0.4 M Tris-HCl buffer pH 9. After the addition of 20 μL 4 mM DTDP, the sample was vortexed and incubated for 5 min in the dark, at room temperature. The absorbance at 324 nm was determined using a Jasco V530 spectrophotometer against a reagent blank. The concentration of protein sulfhydryl groups was calculated using a *N*-acetyl-cysteine standard curve.

#### 3.7.4. Advanced Oxidation Protein Products

The concentration of advanced oxidation protein products (AOPP) was assessed spectrophotometrically [62]. A sample of 200 μL of conveniently diluted total protein extract was mixed with 10 μL 1.16 M potassium iodide and vortexed continuously for 5 min at room temperature. A volume of 20 μL of glacial acetic acid was added and the mixture was vortexed again for 30 seconds. The optical density of samples was determined at a wavelength of 340 nm in a 96 well plate using a Tecan GENios Multireader. The AOPP level of samples was calculated using a chloramine-T standard curve.

#### 3.7.5. Protein Carbonyl Groups Assay

The concentration of carbonyl groups was determined by a method previously described [63]. A volume of 500 μL of appropriately diluted total protein extract was mixed with an equal volume of 10 mM 2,4 dinitrophenylhydrazine prepared in HCl 2 M, vortexed and incubated for one hour at room temperature. Subsequently, 500 μL of 20% TCA were added and the mixture was incubated for 30 min on ice. After centrifugation for 3 min at 13,000 rpm at room temperature, the supernatant was discarded and the pellet was washed two times with 1 mL ethanol: ethyl acetate (1:1) mixture. After 10 minute incubation at room temperature and another centrifugation, the pellet was rendered soluble in 600 μL 1 M NaOH and incubated for 15 min at 37 °C. The absorbance of protein carbonyl was determined at 370 nm against a reagent blank and their concentration was calculated using the molar absorption coefficient of 22,000 M^−1^cm^−1^. The results are expressed in nmoles/mg protein.

#### 3.7.6. Enzyme Activity Assays

Total superoxide dismutase (SOD, EC1.15.1.1) activity was measured using a method, based on NADPH oxidation by the superoxide anion generated from molecular oxygen in a purely chemical reaction in the presence of EDTA, manganese (II) chloride and mercaptoethanol [64]. A control was run with each set of three duplicate samples and the percent inhibition was calculated as sample rate/control rate ×100. One unit of SOD activity was defined as the amount of enzyme which inhibited the oxidation of NADPH compared to control by 50%.

The catalase (CAT, EC 1.11.1.6) activity was assayed by monitoring the disappearance of H_2_O_2_ at 240 nm [65]. The CAT activity was expressed in terms of units. One unit is the amount of enzyme that catalyzed the conversion of one μmole H_2_O_2_ in one minute.

The glucose 6-phosphate dehydrogenase **(**G6PDH, EC 1.1.1.49) activity was measured by recording the increase in absorption at 340 nm due to NADPH formation as a measure of G6PDH activity. The activity was expressed in units [66].

Total glutathione peroxidase (GPx, EC 1.11.1.9) activity was assayed by a method using tert-butyl hydroperoxide and reduced glutathione (GSH) as substrates [67]. The conversion of NADPH to NADP^+^ was followed by recording the changes in absorbance at 340 nm (Perkin Elmer Lambda 35 UV/VIS Spectrometer), and the concentration of NADPH was calculated using a molar extinction coefficient of 6.22 × 10^3^ M^−1^ cm^−1^. One unit of activity was defined as the amount of enzyme that catalyzes the conversion of one μmole of NADPH per minute under standard conditions.

The total glutathione-S-transferase (GST, EC 2.5.1.18) activity was assayed by measuring at 340 nm the rate of 1-chloro-2,4-dinitrobenzene (CDNB) conjugation with GSH [68]. One unit of GST activity was defined as the formation of 1 μmole of conjugated product per minute. The extinction coefficient 9.6 × 10^3^ M^−1^ × cm^−1^ was used for the calculation of CDNB concentration.

The glutathione reductase (GR, EC 1.6.4.2) activity was determined by recording the decrease in absorbance at 340 nm [69]. One unit of GR activity was calculated as one μmole of NADPH consumed per minute under standard conditions.

All enzymatic activities, calculated as specific activities (units/mg protein) were expressed as percentage of controls.

#### 3.7.7. Protein Concentration

The protein concentration (mg/mL) was determined using bovine serum albumin as a standard by Lowry’s method [70].

### 3.8. Western Blot Analysis of Hsp 70

Samples (40 μg) were migrated by SDS PAGE (10% polyacrylamide) and transferred onto 0.45 μm PVDF membrane. In order to detect Hsp 70 and β actin expression, the membranes were developed using anti Hsp 70 monoclonal (2 μg/mL) and anti β actin (2 μg/mL) primary mouse antibodies and the Western Breeze Chromogenic Immunodetection kit with anti mouse secondary antibody coupled with alkaline phosphatase. After transfer, the membrane was incubated for 30 min in blocking solution, then hybridized with the first antibody overnight and washed three times with antibody wash solution. Subsequently, the membrane was incubated with the secondary anti-mouse IgG1 antibody for 30 min, washed two times with distilled water and developed with chromogenic substrate (BCIP/NBT). The immunoreactive bands were visualized and quantified with BioCapt 12.6 software from Vilbert Lourmat.

### 3.9. Statistical Analysis

The values were expressed as means with standard deviations. The differences between the control and quantum dots treated experimental groups were analyzed by Student’s test and validated by confidence intervals using Quattro Pro X3 software (Version 13.0.0.406, Corel, Mountain View, CA, USA, 2005). The results were considered significant only if the *p* value was less than 0.05, and the confidence intervals of the control and samples did not overlap.

## 4. Conclusions

In this study we have investigated the histological and biochemical effects of silicon-based QDs, IP injected in *C. auratus gibelio* over a period of 7 days. All investigated biochemical parameters presented a variation in a time-dependent manner. Also, the expression of Hsp 70 continuously increased up to 7 days.

The histological alterations were not important and signs of tissue regeneration appeared. Taking into account all our data, it appears that a dose of 2 mg silicon-based QDs per kg body weight IP injected in *C. auratus gibelio* caused moderate damage to renal tissue after 7 days of exposure. The oxidative stress induced in this period of time seems to be counteracted by the crucian carp kidney, probably due to the adapted response of the antioxidant system in which Hsp 70 could play a significant role. The signs of nephroneogenesis suggest that crucian carp kidney can cope with oxidative stress under silicon-based QDs.

## Figures and Tables

**Table 1:** The relative values of malondialdehyde and reduced glutathione in the kidney of *Carassius auratus gibelio*, IP injected with silicon-based quantum dots (2 mg/kg body weight).

Time Interval	MDA (nmoles/mg)		GSH (nmoles/mg)	
	
	Control Group	Exposed Group	Control Group	Exposed Group
**1 day**	100 ± 7	197 ± 41 ***	100 ± 6	34 ± 6 ***
**3 days**	100 ± 11	388 ± 108 ***	100 ± 10	40 ± 10 ***
**7 days**	100 ± 8	274 ± 82 ***	100 ± 6	53 ± 8 **

Note: Data are calculated as means ± SD (*n* = 6 in each group at each time point) and expressed as % from controls;

** *p* < 0.01;

*** *p* < 0.001.

**Table 2:** The relative values of proteic thiols, advanced oxidation protein products (AOPP) and protein reactive carbonyl groups (PRCG) in the kidney of *Carassius auratus gibelio* IP injected with silicon-based QDs (2 mg/kg body weight).

	Protein Thiols (nmoles/mg)		AOPP (μmoles/mg)		PRCG (nmoles/mg)	
			
Time Interval	Control Group	Exposed Group	Control Group	Exposed Group	Control Group	Exposed Group
**1 day**	100 ± 19	72 ± 12 ^*^	100 ± 8	111 ± 12	100 ± 22	95 ± 18
**3 days**	100 ± 20	67 ± 15 ^*^	100 ± 13	134 ± 26 ^*^	100 ± 7	222 ± 55 ***
**7 days**	100 ± 18	57 ± 7 **	100 ± 10	110 ± 16	100 ± 27	215 ± 56 ***

Note: Data are calculated as means ± SD (*n* = 6 in each group at each time point) and expressed as % from controls;

** *p* < 0.01;

*** *p* < 0.001.
